# Supplementary material for: Normative brain mapping using scalp EEG and potential clinical application
Source: Sci Rep. 2023 Aug 18;13:13442. doi: 10.1038/s41598-023-39700-7 (PMC10439201; doi:10.1038/s41598-023-39700-7)
Supplement: Supplementary file 1 — Supplementary Information. [file 41598_2023_39700_MOESM1_ESM.docx]

# **Supplementary**

**Supplementary Material 1: Additional information on individual subjects and their EEG**

In the main manuscript we provided descriptive statistics of patients and healthy controls in terms of age, sex (M/F), side (Left/Right), age of onset and duration in Table 1. Here we provide more information on individual subject basis and channels that were removed for individual subject during pre-processing. Note first pathology abbreviations: HS (Hippocampal Sclerosis), EFS (End Folium Sclerosis).

| Patient ID | Age | Age at Onset | Duration | Sex | Side | First Pathology | PET | SPECT | Channels Removed |
| --- | --- | --- | --- | --- | --- | --- | --- | --- | --- |
| 1 | 24 | 15 | 9 | F | Left | Other | yes | no | - |
| 2 | 29 | 15 | 14 | F | Left | HS | yes | no | - |
| 3 | 31 | 13 | 18 | F | Left | HS (EFS) | yes | no | - |
| 4 | 31 | 13 | 18 | F | Left | HS (EFS) | yes | no | - |
| 5 | 33 | 15 | 18 | M | Right | Other | no | yes | - |
| 6 | 19 | 7.5 | 11.5 | F | Left | HS | no | no | - |
| 7 | 42 | 3 | 39 | F | Left | Other | yes | no | TP10, O2 |
| 8 | 24 | 19 | 5 | M | Right | Other | yes | no | - |
| 9 | 47 | 14 | 33 | M | Right | Other | no | yes | - |
| 10 | 25 | 9 | 16 | F | Left | HS | no | no | F7, F8 |
| 11 | 38 | 30 | 8 | F | Right | HS | no | no | - |
| 12 | 48 | 4 | 44 | M | Left | HS | no | no | - |
| 13 | 47 | 9 | 38 | F | Left | HS | no | no | - |
| 14 | 57 | 5 | 52 | M | Right | Other | no | no | O1, O2, Oz |
| 15 | 33 | 2 | 31 | M | Left | HS | no | no | - |
| 16 | 26 | 7 | 19 | M | Left | HS | no | no | - |
| 17 | 35 | 18 | 17 | M | Right | Other | no | yes | TP9 |
| 18 | 31 | 24 | 7 | F | Left | Other | no | no | - |
| 19 | 32 | 10 | 22 | M | Left | HS | yes | no | Fp1, Fp2, F8 |
| 20 | 45 | 17 | 28 | F | Right | HS (EFS) | yes | no | P7, C3 |
| 21 | 19 | 3 | 16 | F | Left | HS (EFS) | no | no | - |
| 22 | 37 | 32 | 5 | F | Right | Other | yes | no | Fp2 |

**Table S1: Additional details of patient data**

**Table S2: Details of healthy control data**

| Control ID | Age | Sex | Channels Removed |
| --- | --- | --- | --- |
| 1 | 28 | F | - |
| 2 | 39 | M | - |
| 3 | 49 | F | - |
| 4 | 29 | F | - |
| 5 | 41 | M | - |
| 6 | 35 | M | - |
| 7 | 28 | M | - |
| 8 | 30 | M | - |
| 9 | 30 | F | - |
| 10 | 33 | F | - |
| 11 | 28 | M | T7 |
| 12 | 22 | M | - |
| 13 | 30 | M | - |
| 14 | 28 | M | T8 |
| 15 | 34 | M | - |
| 16 | 25 | F | T8 |
| 17 | 33 | M | - |

**Supplementary Material 2: Estimating normative map robustness – Epoch 3**

In the main manuscript we presented correlation and standard deviation graphs of normative map 1 vs map 2. Here we present additional figures that involve map 3 and their respective correlation results


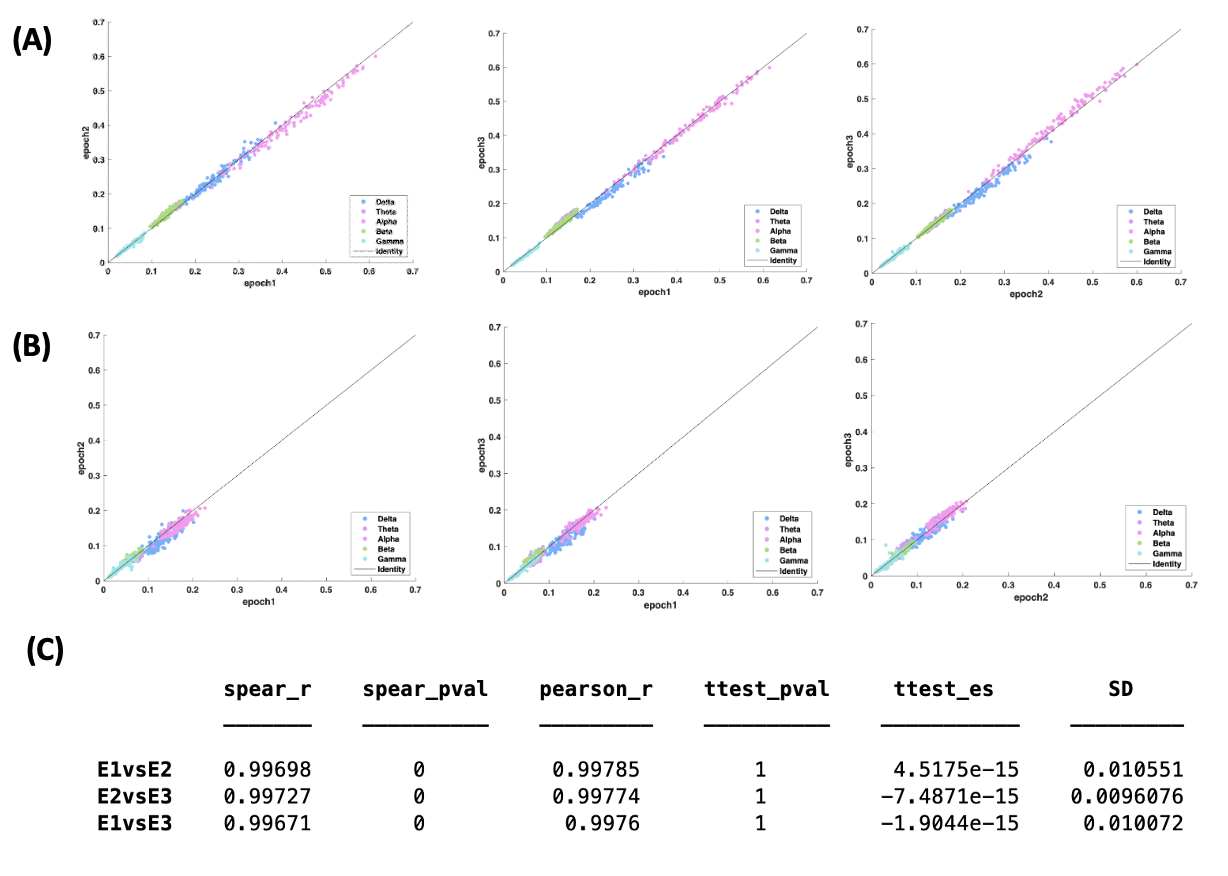


**Figure S2: Reproducibility analysis of normative map robustness across individual epochs.** Relative ROI band power averaged across the healthy subjects for the five individual frequency bands (from top to bottom: delta, theta, alpha, beta, gamma). ROI band power calculated for three, 30-second, non-overlapping epochs: mean relative band power (A), and standard deviation (B).

**Supplementary Material 3: Normative map consistency across parcellations**

In the main text we adapted the Lausanne parcellation of scale 60 with 114 regions of interest. Here we illustrate similarity Comparison of scalp normative maps across three alternative parcellations. Resolution sizes of the following include: 68, 219 and 448 regions, respectively.


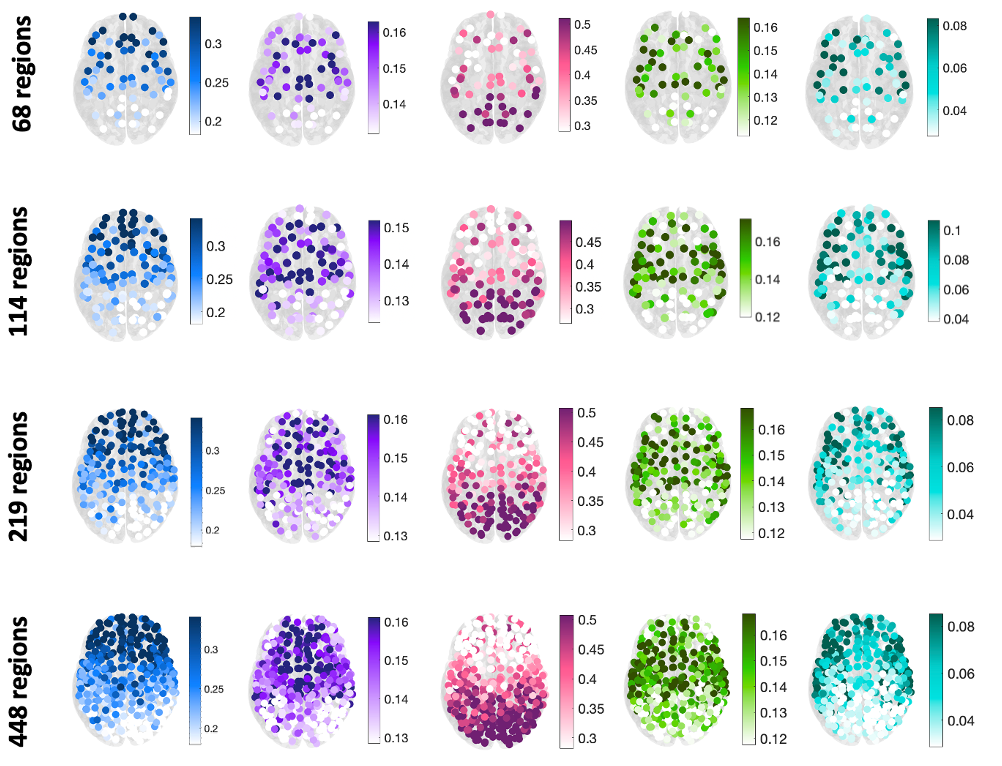


**Figure S3: Replication of normative map results across four different resolution parcellations, across five frequency bands.** Relative ROI band power averaged across the healthy subjects for the five individual frequency bands (from left to right: delta, theta, alpha, beta, gamma), and across different Lausanne resolution scales (from top to bottom: 68, 114, 219, 448). The power density colour scales are normalised to each band’s power range.

**Supplementary Material 4: Similarity of normative maps across samples**

Here we present suggestive results that a small sample size is sufficient to produce reliable normative brain maps. We investigated the similarity of normative maps across different number of samples and computed the spearman correlations.

To do this, in each permuted group we randomly selected two independent sets of *n* healthy controls and correlated their brain maps. In simulated groups of sample size two and above, we averaged the respective number of healthy control maps in each group, before correlating. We ran a 1000 permutations per sample size and plotted group averages with error bars, with no subject overlap in each loop iteration.

We observe that similarity rate increases with a greater number of samples per group, with reducing error bars. Sample size of six and above have a mean and standard deviation above Spearman rho=0.9

In the main text, we used 17 healthy controls to compute the normative maps. Here, similarity results indicate that a small sample size of under 10 subjects is sufficient to produce a robust normative map.


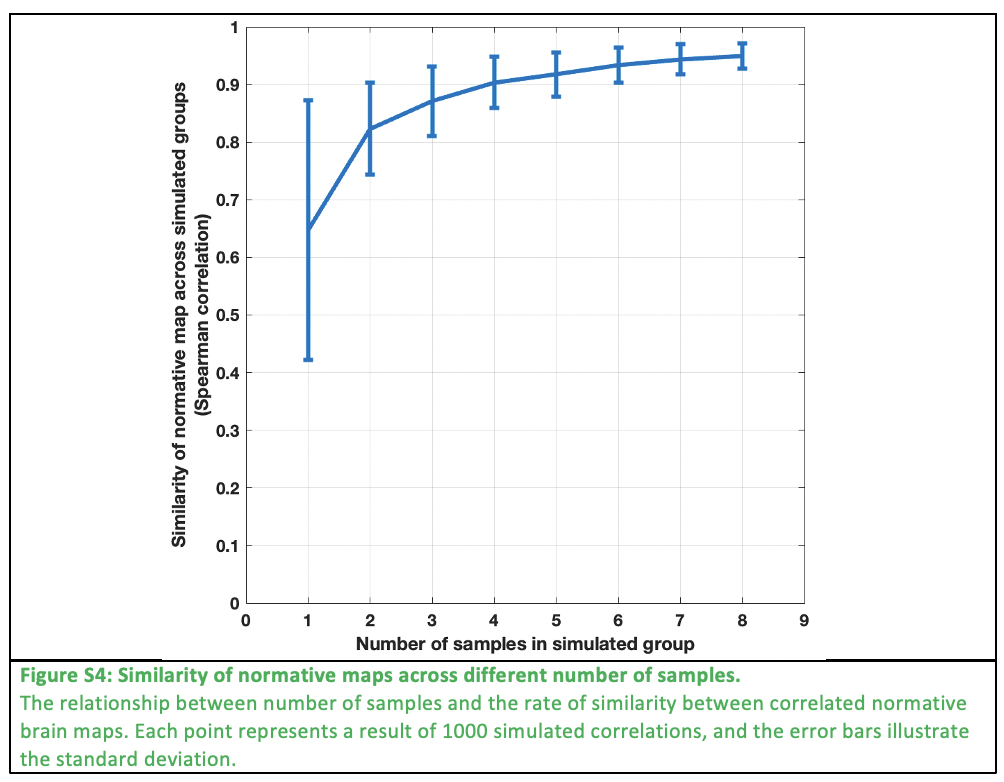


**Figure S4: Similarity of normative maps across different number of samples.**

The relationship between number of samples and the rate of similarity between correlated normative brain maps. Each point represents a result of 1000 simulated correlations, and the error bars illustrate the standard deviation.

**Supplementary Material 5: Estimating the performance of prediction of laterality.**

In the following analysis we investigate the performance of our abnormality metric to predict laterality in temporal lobe epilepsy, using area under curve (AUC) analysis. We hypothesised that the sum of maximum absolute z-scores across all temporal regions is greater in the ipsilateral hemisphere.

To quantify the prediction of laterality, we totalled the max absolute z-scores in each hemisphere and subtracted the sums independently across each patient. Positive values indicate greater left-sided abnormality, whilst negative values indicate the opposite. Fig S5A sows these values across patients. One-tailed Wilcoxon signed rank test found significantly greater values in left TLE than right TLE as expected (p=0.023).

We then performed AUC analysis (where the curve is the receiver operating characteristic), shown in Fig S5B. We imported information regarding which hemisphere was resected in individual patients and the predictive outcome value from the laterality equation. The resulting AUC is 0.7500

Overall, the results are suggestive of good performance by the laterality predictor.


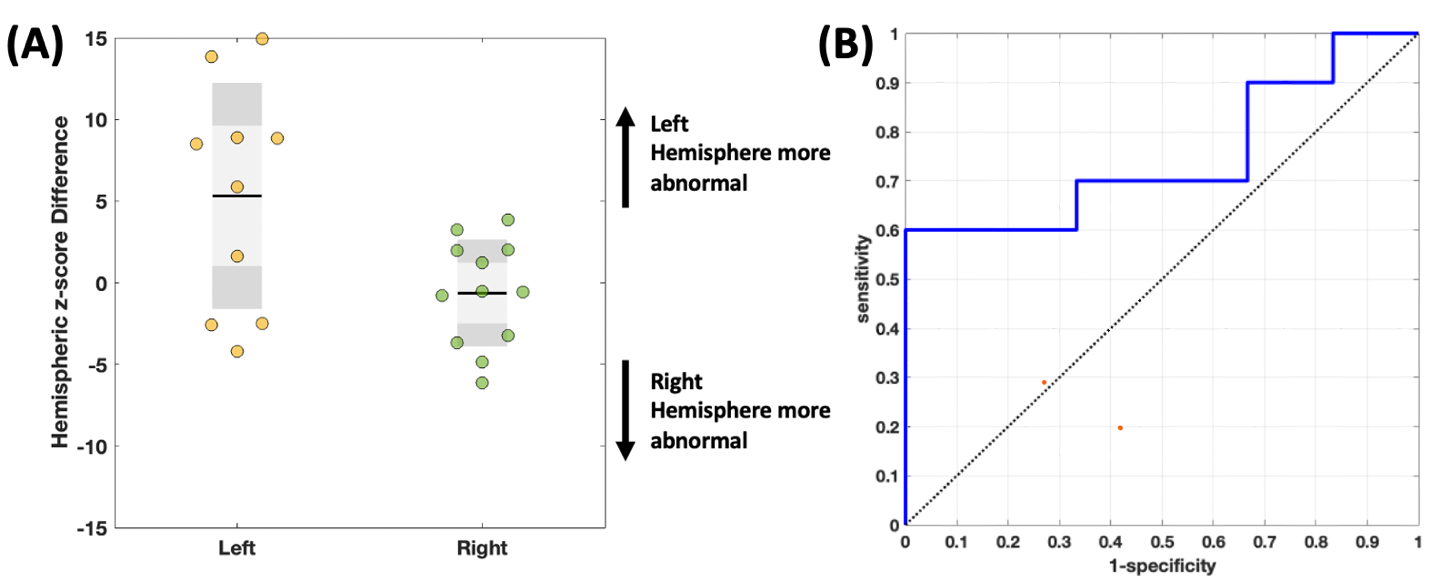


**Figure S5: Performance of laterality predictor.** (A) Univariate scatter plot based on the outcome values has the interpretation that more positive is more abnormal in the left hemisphere, whereas more negative is more abnormal in the right hemisphere. (B) Receiver operating characteristic (ROC) curve, illustrating the performance of the laterality predictor using classification thresholds.
